# Inducing rapid telomere irreparable damage in telomerase-expressing cancers

**DOI:** 10.18632/oncotarget.26317

**Published:** 2018-11-09

**Authors:** Gao Zhang, Jerry W. Shay

**Affiliations:** UT Southwestern Medical Center, Department of Cell Biology, Dallas, TX, USA

**Keywords:** telomeres, telomerase, melanoma, lung cancer, brain cancer

Telomeres are the protective structures at the ends of linear chromosomes that progressively shorten each time when a cell divides which is in part caused by the end-replication problem. Telomeres are protected by a series of six proteins termed the shelterin complex that prevents the linear chromosome ends from being recognized as DNA double-strand breaks that otherwise activates damage repair responses [1]. Thus, in the absence of a mechanism that is in place to maintain telomeres, telomeres in cells will be progressively shortened, leading to a phenomenon known as replicative senescence. How then do advanced cancer cells maintain telomeres? The answer is that telomerase (a cellular ribonucleoprotein enzyme complex with reverse transcriptase activity) is expressed in almost all human cancers and provides cancer cells with proliferative immortality while most human tissues remain telomerase silent [2]. Thus, telomerase is a hallmark of cancer and an almost universal target for cancer therapy. However, direct telomerase inhibitors have not progressed well in clinical development [3]. Part of the problem is that even robust and direct inhibition of telomerase requires a significant lag period between the time of enzyme inhibition and the time to trigger biological effects. Thus, anti-telomerase treatments require long-term and progressive telomere shortening that eventually leads to apoptosis/cell death in order to achieve tumor shrinkage. In almost all clinical trials, serious toxicities occur and require patients to stop therapy, and this in turn results in the rapid regrowth of telomeres [4]. Thus, novel strategies are needed to target telomerase in cancer cells. The approach that aims to eliminate the long lag period from initiation of treatment to tumor shrinkage is the subject of this editorial.

**Figure 1:**
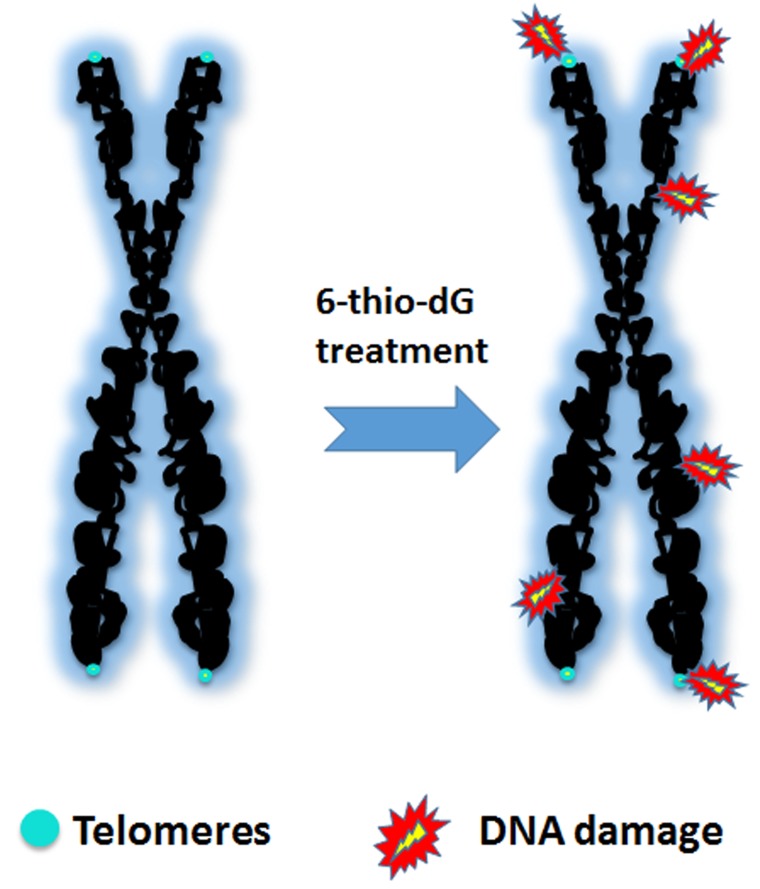
Human telomeres are TTAGGG repetitive DNA sequences at the ends of linear chromosomes that are protected by shelterin proteins from being recognized as DNA damage However, during DNA synthesis the telomeres must unfold so the free ends are accessible to the replication machinery. In the presence of 6-thio-dG telomerase preferentially incorporates 6-thio-guanosine triphosphate into the telomeric repeats resulting in DNA damage and cell death, but only in telomerase expressing cells.

In 1977 clinical trials focusing on cancer with a compound called, β-2'-deoxythioguanosine, demonstrated that the compound was well tolerated and that remissions can be achieved in acute leukemia [5]. While there were some hematological toxicities at the doses used, the conclusion was that β-2'-deoxythioguanosine warranted further study to improve cancer patient outcomes [5]. The metabolic studies with β-2'-deoxythioguanosine indicated it was similar to 6-thioguanine. 6-thioguanine is a compound that continues to be used to the present, but the β-2'-deoxythioguanosine (a nucleoside analogue) was not pursued until recently [6]. In 2018, four publications [7–10] demonstrated the efficacy of β-2'-deoxythioguanosine (now referred to as 6-thio-2'-deoxyguanosine or 6-thio-dG) across a variety of cancer types. 6-thio-dG appears to work by a novel mechanism that results in telomere uncapping but this only occurs in telomerase-positive cells [6]. 6-thio-dG preferentially uses telomerase to incorporate an altered sequence into the TTAGGG repeats of telomeres that results in the formation and accumulation of telomere-induced dysfunctional foci (TIF) (Figure 1), leading to rapid cell death [6]. TIFs do not occur with 6-thioguanine. The effective daily dose of 6-thio-dG is in the range of ~2.5-5 mg/kg which is less toxic in 129S2 wild-type mice compared to that of 6-thioguanine [6].

Two of these recent reports indicated that 6-thio-dG was effective in treating primary melanoma as a monotherapy and also in targeted therapy-refractory melanoma [7-8]. In the report by Reyes-Uribe and coworkers [7], 6-thio-dG was effective in treating NRAS-mutated melanomas as a monotherapy since patient have limited therapeutic options and poor prognosis. They next found combining 6-thio-dG with the mitochondrial inhibitor, gamitrinib, more effectively suppressed the viability of NRAS-mutant melanoma indicating a robust dependency of NRAS-mutant melanoma on telomerase and metabolic function as well as providing a proof of principal for new combination therapeutic strategies to combat this currently untreatable type of melanoma. In the second report on melanoma, Zhang and coworkers [8] demonstrated the efficacy of 6-thio-dG both *in vitro* and *in vivo* of melanomas that are resistant to targeted therapies as well as in tumor cells derived from patients who were refractory to immune checkpoint inhibitors. These results show that directly targeting aberrant telomerase in melanomas with 6-thio-dG is a viable therapeutic approach to prolong disease control and to overcome therapy resistance.

The third report examined the effects of 6-thio-dG in both targeted therapy resistant non-small cell lung cancer (NSCLC) and chemotherapy resistant NSCLC [9]. Mender and coworkers [9] showed that NSCLC cells resistant to the first-generation EGFR inhibitor, erlotinib, remained highly sensitive to 6-thio-dG even at low nanomolar concentrations. Similar results with 6-thio-dG were obtained with EGFR mutant tumor cells resistant to osimertinib. In addition, Mender et al [9] demonstrated that NSCLC cells resistant to both paclitaxel and carboplatin were sensitive to 6-thio-dG. Importantly, this report showed that 6-thio-dG was orally effective leading to TIFs and tumor shrinkage in xenograft models.

The fourth recent report from Sengupta and co-workers [10] tested the effects of 6-thio-dG on treating therapy-resistant pediatric brain tumors. Brain tumors remain the leading cause of cancer-related death in children. This report examined high-risk meduloblastomas and high-grade gliomas for the effects of 6-thio-dG. They observed that 6-thio-dG delayed tumor growth and increased DNA damage at telomeres which resulted in increased apoptosis without affecting normal telomerase silent fibroblasts. Most importantly, they demonstrated in orthotopic xenograft models that 6-thio-dG crosses the blood-brain barrier and specifically targets tumor cells.

In summary, while there have been impressive results with immune checkpoint inhibitors for the first-line treatment of multiple types of human cancers, there remains an urgent clinical need for identifying novel and effective therapeutic targets in order to overcome acquired drug resistance and to treat patients who do not respond to immune checkpoint inhibitors and other targeted therapies due to intrinsic drug resistance. These recent publications exploiting the efficacy of 6-thio-dG suggest that conventional approaches to target cancer in combination or sequentially with new targets such as telomeres may have great utility in the clinical setting.
